# Association between serum vitamin D levels and polycystic ovary syndrome: a cross-sectional study

**DOI:** 10.3389/fnut.2026.1728823

**Published:** 2026-01-26

**Authors:** Yuling Liu, Jia Fang, Xiaoqing An, Meng Zheng, Huanhuan Liu, Yaoyao Zhang, Jiaojie Chen, Guilin Jiang

**Affiliations:** 1 Hospital Affiliated to Jiangxi University of Chinese Medicine, Nanchang, China; 2 Jiangxi University of Chinese Medicine, Nanchang, China

**Keywords:** dose–response relationship, polycystic ovary syndrome, risk factors, subgroup analysis, vitamin D

## Abstract

**Objective:**

Polycystic ovary syndrome (PCOS) is a common endocrine and metabolic disorder among women of reproductive age, significantly increasing the risk of obesity, insulin resistance, and reproductive dysfunction. Vitamin D (VD) plays an important role in metabolic regulation, immune modulation, and inflammatory responses, but its association with PCOS risk remains controversial. This study aimed to evaluate the association between serum VD levels and the risk of PCOS, and to explore potential modifying effects across different populations.

**Methods:**

A total of 1,397 female participants were included. Multivariate logistic regression models were used to assess the association between serum VD levels and PCOS, while restricted cubic spline (RCS) regression was applied to examine the dose–response relationship. Subgroup analyses were further conducted to explore the potential modifying effects of age, BMI, and marital status on the association between VD and PCOS risk.

**Results:**

Serum VD levels were significantly and inversely associated with PCOS risk (*P* < 0.001), and this association remained robust after adjustment for demographic, behavioral, and comorbid factors (OR = 0.58, 95% CI: 0.44–0.76). The RCS analysis revealed a linear dose–response relationship between serum VD levels and PCOS risk, with higher VD concentrations associated with a progressively lower risk of PCOS. Subgroup analyses indicated that this inverse association was more pronounced among women aged ≥ 40 years, those with BMI ≥ 30, and individuals who were divorced, separated, or widowed, while no significant interaction was observed in other subgroups.

**Conclusion:**

Serum VD level is an independent protective factor against PCOS, and higher concentrations are associated with a lower risk of PCOS, particularly among older, obese, and certain marital status subgroups. This study provides epidemiological evidence supporting the potential value of VD in the prevention and intervention of PCOS, and suggests that future research should further explore its mechanistic pathways and individualized intervention strategies.

## 1 Introduction

Polycystic ovary syndrome (PCOS) is one of the most common endocrine and metabolic disorders among women of reproductive age, clinically characterized by irregular menstruation, hyperandrogenism, and polycystic ovarian morphology (1). Epidemiological studies indicate that the global prevalence of PCOS ranges from approximately 6–20%, with substantial variation due to differences in diagnostic criteria and population characteristics (2). As a complex multifactorial disorder, PCOS is closely associated with infertility and significantly increases the risk of obesity, insulin resistance, type 2 diabetes, and cardiovascular diseases, thereby severely affecting women’s reproductive health and overall quality of life (3, 4). Therefore, identifying its risk factors and potential intervention targets has become a major focus of current research.

In recent years, VD (VD) has attracted attention due to its wide-ranging physiological roles beyond bone metabolism (5). In addition to its classical functions in regulating calcium-phosphate homeostasis and skeletal health, VD plays an important role in insulin secretion and sensitivity, immune modulation, and inflammatory responses (5). Previous studies have suggested that VD deficiency or insufficiency is prevalent among women with PCOS and may be closely linked to metabolic disturbances, menstrual irregularities, and reproductive dysfunction (6, 7). However, epidemiological evidence regarding the association between VD and PCOS remains somewhat controversial, and mechanistic studies require further elucidation (8). Based on this, the present study aims to systematically investigate the relationship between VD levels and the development of PCOS, and to analyze differences across populations with varying metabolic and reproductive characteristics. By integrating clinical and mechanistic evidence, this study seeks to provide new scientific insights into the potential role of VD in the prevention and management of PCOS, and to inform strategies for improving women’s health and implementing precision interventions.

## 2 Materials and methods

### 2.1 Study design

To enhance methodological transparency and reduce potential bias, several strategies were implemented in the study design and analysis. First, this study was based on a single-center hospital electronic health system (HIS) rather than a population-based survey; therefore, survey weights and complex sampling designs were not applicable. To minimize selection bias, all eligible patients with complete exposure, outcome, and covariate data during the study period were consecutively included according to predefined inclusion and exclusion criteria. Second, multiple covariate adjustments were applied in multivariable models, including demographic, lifestyle, reproductive, physiological, and biochemical factors, to reduce potential confounding. Third, consistent diagnostic criteria and validated laboratory assays were used for exposure and outcome measurements to limit misclassification bias. Finally, subgroup and sensitivity analyses were conducted to evaluate the robustness of the findings and explore potential residual confounding.

### 2.2 Study population

**FIGURE 1:**
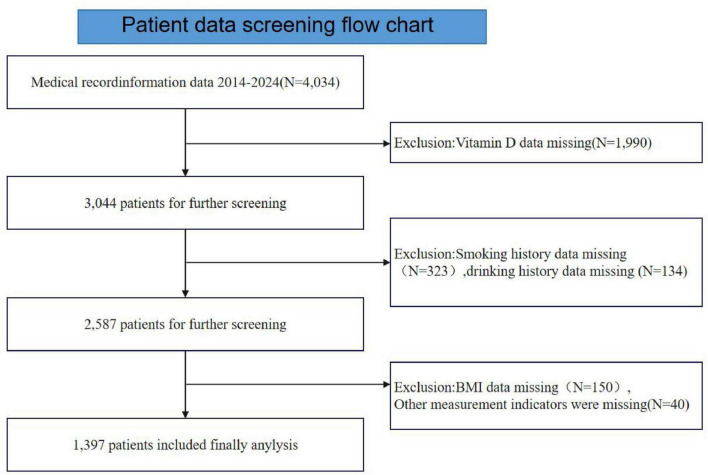
The flow diagram of the study participants.

This study is a retrospective analysis, with data derived from patients at the Affiliated Hospital of Jiangxi University of Traditional Chinese Medicine between June 2014 and December 2024. The study population included individuals with complete records in the hospital electronic health system (HIS), initially screening 4,034 patients. According to exclusion criteria, patients with missing serum VD data (*N* = 1,990) were removed from the study. In addition, patients were excluded due to missing covariate information, including missing smoking data (*N* = 323), missing alcohol consumption data (*N* = 134), and missing BMI data (*N* = 190). Ultimately, a total of 1,397 participants were included in this study. The complete participant screening flow is shown in Figure 1. This study was conducted in accordance with the principles of the Declaration of Helsinki and was approved by the Ethics Committee of the Affiliated Hospital of Jiangxi University of Traditional Chinese Medicine (Ethics approval number: JZFYLL202402230018). This study did not undertake a formal sample size calculation; all eligible cases were included in the analysis from the existing medical records database. The study population included ALL individuals with complete records in the hospital electronic health system (HlS).

### 2.3 Study variables

#### 2.3.1 Diagnosis of polycystic ovary syndrome

In this study, the diagnosis of PCOS was based on the Rotterdam criteria jointly proposed by the 2003 European Society of Human Reproduction and Embryology (ESHRE) (9) and the American Society for Reproductive Medicine (ASRM) (3). After excluding other conditions that could cause menstrual irregularities or hyperandrogenism—such as thyroid dysfunction, hyperprolactinemia, congenital adrenal hyperplasia, Cushing’s syndrome, and androgen-secreting tumors—PCOS was diagnosed if at least two of the following three criteria were met: (1) Oligo-ovulation or anovulation; (2) Clinical or biochemical signs of hyperandrogenism; (3) Polycystic ovarian morphology, defined as one or both ovaries containing ≥ 12 follicles with a diameter of 2–9 mm, or ovarian volume > 10 mL. The Rotterdam diagnostic criteria were applied consistently across all participants, and all clinical, biochemical, and ultrasonographic assessments were performed according to standardized hospital protocols to ensure diagnostic consistency.

#### 2.3.2 Serum vitamin D

Based on previous studies, serum VD deficiency was defined as a concentration < 50.00 nmol/L, whereas a serum VD concentration ≥ 50.00 nmol/L was considered normal (10). Serum vitamin D concentrations were measured using standardized and validated laboratory assays in the hospital clinical laboratory, with regular internal quality control procedures in place to ensure measurement reliability. The results are shown in Table 3.

#### 2.3.3 Covariates

Covariates were selected *a priori* based on established epidemiological evidence and biological plausibility. Specifically, demographic characteristics, lifestyle factors, reproductive history, physiological indicators, and biochemical markers were selected based on their established epidemiological and biological relevance to both serum vitamin D levels and PCOS risk. These covariates may influence vitamin D metabolism, inflammatory and metabolic status, reproductive endocrine function, and health behaviors, and therefore represent important potential confounders.

Covariates included demographic characteristics, lifestyle factors, reproductive history, physiological indicators, and biochemical markers. Demographic variables comprised age, educational level, and marital status, which reflect age-related metabolic and endocrine changes as well as socioeconomic and psychosocial factors influencing health behaviors, sunlight exposure, and healthcare access. Age was categorized as < 40 and ≥ 40 years, and marital status as married/living with partner, never married, or widowed/divorced/separated. Lifestyle factors included smoking and alcohol consumption, both of which are associated with altered vitamin D metabolism, inflammation, endocrine function, and metabolic risk. Smoking was defined as having smoked ≥ 100 cigarettes in a lifetime, and alcohol consumption as drinking ≥ 12 alcoholic beverages per year. Reproductive history was assessed by pregnancy status (ever vs. never pregnant), reflecting cumulative hormonal exposure and reproductive endocrine function that may confound the association of interest. Physiological indicators included BMI, hypertension, and diabetes, representing key components of metabolic dysfunction and systemic inflammation closely linked to vitamin D bioavailability and disease risk. BMI was categorized as < 25, 25–30, and ≥ 30 kg/m^2^. Hypertension and diabetes were defined using standard diagnostic criteria based on self-report, clinical measurements, or medication use. Biochemical markers included serum vitamin D, C-reactive protein, total testosterone, and glutathione, capturing inflammatory, oxidative stress, and hormonal status. All biochemical variables were analyzed as continuous measures to preserve information and reflect their original scales.

### 2.4 Statistical analysis

Participants’ baseline characteristics were classified according to the presence or absence of PCOS. Continuous variables were expressed as mean ± standard deviation (mean ± SD), and categorical variables were expressed as percentages (%). Differences between categorical variables were assessed using the chi-square (χ^2^) test, while differences between continuous variables were evaluated using the Kruskal–Wallis test (11). A two-sided *p*-value < 0.05 was considered to indicate statistical significance.

Following descriptive analyses, multivariate logistic regression models were used to examine the association between serum VD levels and the risk of PCOS, quantified as odds ratios (ORs) with 95% confidence intervals (CIs). Three models were established to sequentially control for potential confounders: Based on the predefined covariates, three progressively adjusted logistic regression models were constructed. Model 1 was unadjusted. Model 2 adjusted for demographic and lifestyle factors, including age (< 40 or ≥ 40 years), educational level, marital status (married/living with partner, never married, widowed/divorced/separated), smoking status, and alcohol consumption. Model 3 further adjusted for reproductive history (ever pregnant or never pregnant), physiological indicators including body mass index (BMI; < 25, 25–30, or ≥ 30 kg/m^2^), hypertension, and diabetes, as well as biochemical markers including CRP, total testosterone, and glutathione (GSH), based on Model 2. Serum VD concentration (nmol/L) was analyzed as the exposure variable across all models. Additionally, restricted cubic spline (RCS) models were applied to evaluate the non-linear dose–response relationship between serum VD levels and PCOS risk, adjusting for the same covariates included in Model 3 (11). In addition, subgroup analyses were conducted to assess the robustness of associations and potential heterogeneity, exploring the modifying effects of age, marital status, BMI, smoking, alcohol consumption, and hypertension on the relationship between VD and PCOS risk (12). Furthermore, sensitivity analyses were performed to evaluate the stability of the main findings. These analyses included repeating the multivariable models using alternative categorizations of serum vitamin D status based on different cutoff values reported in previous literature, as well as excluding participants with major metabolic comorbidities. The overall direction and magnitude of the associations remained consistent, indicating the robustness and reliability of the results.

## 3 Results

### 3.1 Baseline characteristics of the study participants

A total of 1,397 women aged ≥ 20 years were included, of whom 590 (42.2%) were diagnosed with PCOS (Table 1). No significant difference was observed in age distribution between the PCOS and non-PCOS groups (*P* = 0.089). Compared with non-PCOS participants, those with PCOS were less likely to be married or cohabiting (55.1% vs. 68.7%) and more likely to be widowed, divorced, or separated (18.3% vs. 9.7%; *P* < 0.001). Higher BMI (≥ 30 kg/m^2^) and smoking prevalence were also more common in the PCOS group (42.9% vs. 34.2% and 46.8% vs. 35.8%, respectively; both *P* < 0.001), whereas drinking status, hypertension, and diabetes did not differ significantly. Biochemically, women with PCOS had lower serum VD (51.87 ± 25.50 vs. 64.45 ± 26.03 nmol/L; *P* < 0.001) and glutathione levels (4.63 ± 1.05 vs. 5.01 ± 0.98 μmol/L; P < 0.001), but higher testosterone (0.81 ± 0.36 vs. 0.48 ± 0.21 ng/mL; *P* < 0.001) and CRP levels (4.4 ± 3.1 vs. 3.5 ± 2.4 mg/L; P < 0.001). Pregnancy history differed significantly, with fewer PCOS participants having ever been pregnant (63.6% vs. 80.6%; P < 0.001). Overall, PCOS was associated with adverse demographic, lifestyle, and biochemical profiles, including higher BMI, smoking, testosterone, and CRP levels, and lower VD and glutathione levels.

**TABLE 1:** Basic characteristics of participants.

Characteristic	Overall (*N* = 1397)	Non-PCOS (*N* = 807)	PCOS (*N* = 590)	*P*-value
Age (years)				0.089^a^
<40	973 (69.65%)	577 (71.50%)	396 (67.12%)	
≥40	424 (30.35%)	230 (28.50%)	194 (32.88%)	
Marital status, n (%)				<0.001^a^
Married/Living with partner	879 (62.92%)	554 (68.65%)	325 (55.08%)	
Never married	332 (23.77%)	175 (21.69%)	157 (26.61%)	
Widowed/Divorced/Separated	186 (13.31%)	78 (9.67%)	108 (18.31%)	
BMI, n (%)				<0.001^a^
<25	476 (34.07%)	307 (38.04%)	169 (28.64%)	
25–30	392 (28.06%)	224 (27.76%)	168 (28.47%)	
≥30	529 (37.87%)	276 (34.20%)	253 (42.88%)	
Smoking status, n (%)				<0.001^a^
No	832 (59.56%)	518 (64.19%)	314 (53.22%)	
Yes	565 (40.44%)	289 (35.81%)	276 (46.78%)	
Drinking status, n (%)				0.800^a^
No	514 (36.79%)	300 (37.17%)	214 (36.27%)	
Yes	883 (63.21%)	507 (62.83%)	376 (63.73%)	
Hypertension, n (%)				0.200^a^
No	1123 (80.39%)	659 (81.66%)	464 (78.64%)	
Yes	274 (19.61%)	148 (18.34%)	126 (21.36%)	
Diabetes, n (%)				0.700^a^
No	1333 (95.42%)	772 (95.66%)	561 (95.08%)	
Yes	64 (4.58%)	35 (4.34%)	29 (4.92%)	
Serum vitamin D (nmol/L)	59.14 (26.54)	64.45 (26.03)	51.87 (25.50)	<0.001^b^
Pregnancy history, n (%)				<0.001^a^
Ever pregnant	1025 (73.34%)	650 (80.55%)	375 (63.56%)	
Never pregnant	372 (26.66%)	157 (19.45%)	215 (36.44%)	
CRP (mg/L)	3.9 (2.8)	3.5 (2.4)	4.4 (3.1)	<0.001^b^
Testosterone (ng/mL)	0.62 (0.32)	0.48 (0.21)	0.81 (0.36)	<0.001^b^
Glutathione (μmol/L)	4.85 (1.02)	5.01 (0.98)	4.63 (1.05)	<0.001^b^

Continuous variables are presented as mean (standard deviation), and categorical variables as number (percentage).

^a^*P*-values were calculated using Pearson’s Chi-squared test.

^b^*P*-values were calculated using the Kruskal–Wallis test.

### 3.2 Association between serum VD Levels and PCOS Risk

**FIGURE 2:**
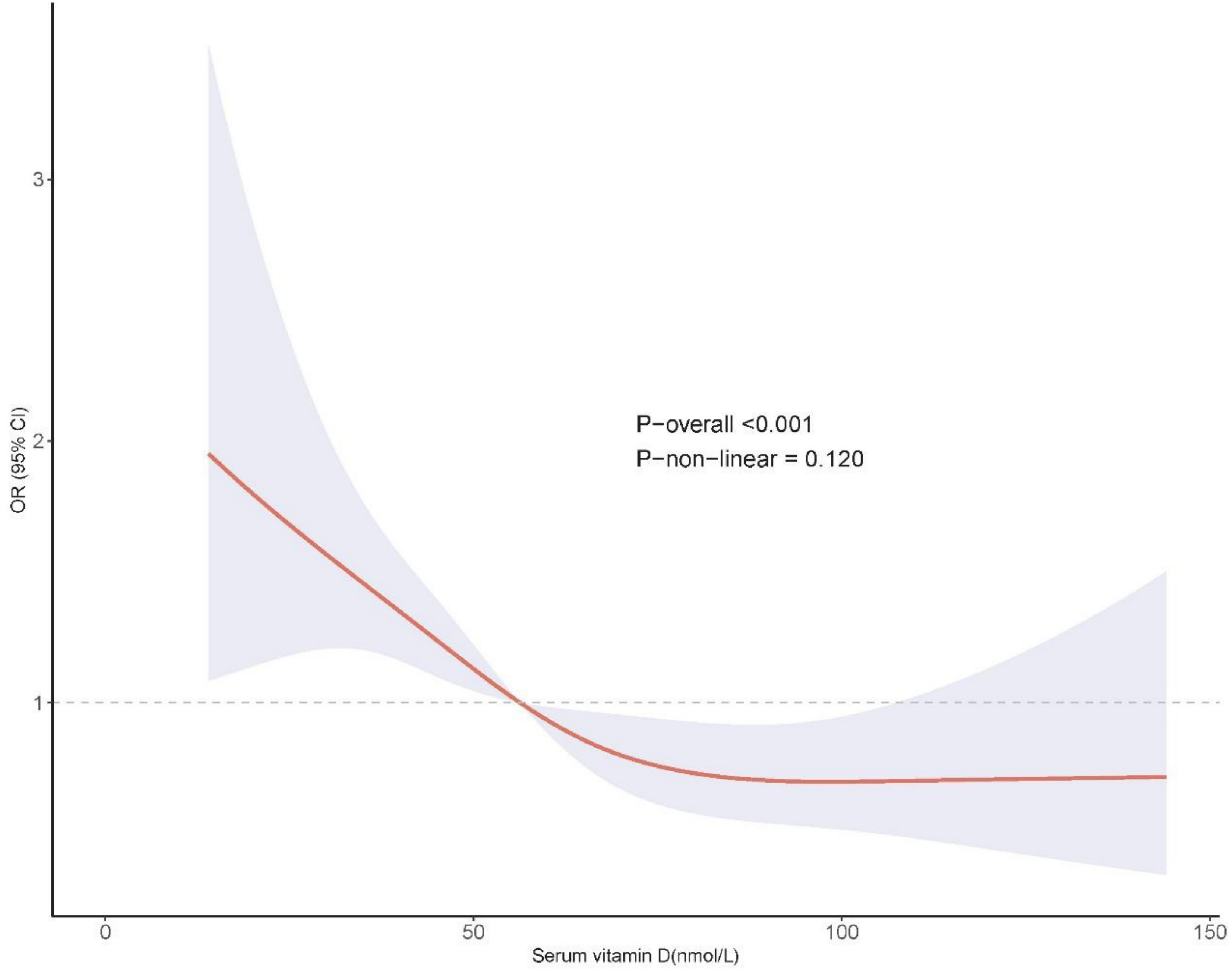
The RCS curve showed a dose response between serum VD and PCOS risk.

The relationship between serum VD levels and the risk of polycystic ovary syndrome (PCOS) is summarized in Table 2. In the unadjusted model (Model 1), each 1 nmol/L increase in serum VD was associated with a 2% reduction in PCOS risk (OR = 0.98, *P* < 0.001). After sequential adjustment for demographic and lifestyle factors in Model 2 and additional comorbidities in Model 3, the inverse association was slightly attenuated but remained statistically significant (Model 3: OR = 0.99, *P* < 0.001), suggesting that higher VD levels may independently reduce the risk of PCOS. When serum VD was analyzed as a categorical variable, participants with normal VD levels (≥ 50 nmol/L) had a 42% lower risk of PCOS compared with those with VD deficiency (< 50 nmol/L) in the fully adjusted model (Model 3: OR = 0.58, 95% CI: 0.44–0.76, *P* < 0.001). Restricted cubic spline (RCS) analysis (Figure 2) revealed no evidence of a significant non-linear association between serum VD levels and PCOS risk (*P* for non-linearity = 0.120). The results showed a consistent downward trend in PCOS risk as VD levels increased, supporting a dose-response relationship. Overall, these findings indicate that higher serum VD levels are independently associated with a lower risk of PCOS, highlighting the potential protective role of VD in reproductive health. The results are shown in Table 2.

**TABLE 2:** Association between serum vitamin VD and PCOS risk.

Characteristic	Model 1 OR (95% CI)	*p*-value	Model 2 OR (95% CI)	*p*-value	Model 3 OR (95% CI)	*p*-value
Serum vitamin D (nmol/L)	0.98 (0.98–0.98)	<0.001	0.99 (0.98–1.00)	<0.001	0.99 (0.98–1.00)	<0.001
**Serum vitamin D subgroups**						
Deficient (< 50 nmol/L)	Reference	–	Reference	–	Reference	–
Normal (≥ 50 nmol/L)	0.37 (0.30–0.47)	<0.001	0.58 (0.45–0.76)	<0.001	0.58 (0.44–0.76)	<0.001

Model 1: Unadjusted. Model 2: Adjusted for demographic and lifestyle factors, including age, educational level, marital status, smoking status, and alcohol consumption. Model 3: Further adjusted for reproductive history (pregnancy status), physiological indicators (body mass index, hypertension, diabetes), and biochemical markers (C-reactive protein, total testosterone, and glutathione), based on Model 2.

### 3.3 Subgroup analysis

**FIGURE 3:**
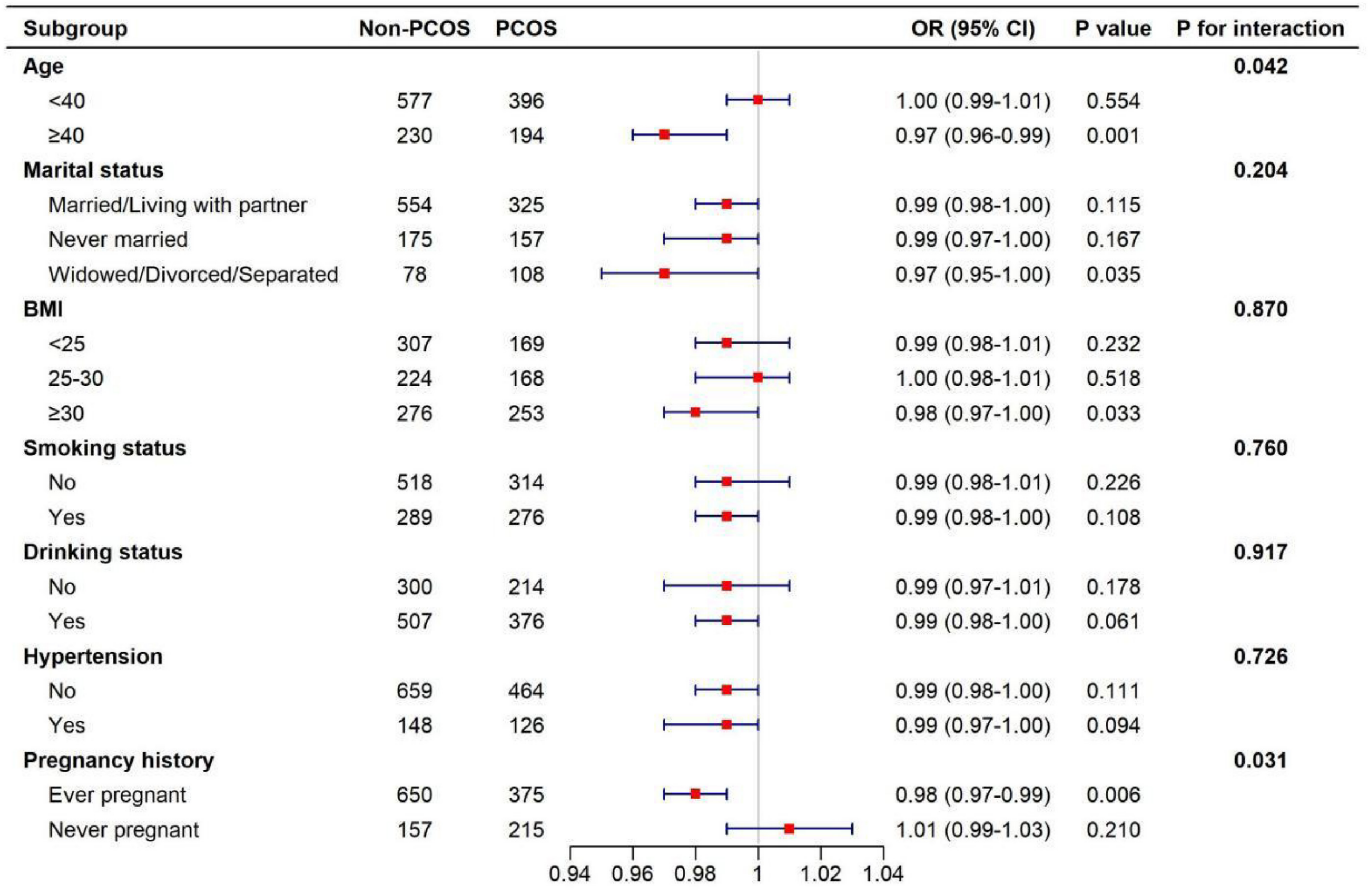
Subgroup analysis of the association between serum VD and PCOS risk.

The subgroup analyses demonstrated several patterns of effect modification in the association between serum VD levels and PCOS. A statistically significant interaction was observed for age (*P* for interaction = 0.042). Among women aged < 40 years, serum VD was not significantly associated with PCOS (OR = 1.00, 95% CI: 0.99–1.01, *P* = 0.554), whereas a significant inverse association was observed among those aged ≥ 40 years (OR = 0.97, 95% CI: 0.96–0.99, *P* = 0.001). No significant interaction was detected for marital status (P for interaction = 0.204), although women who were widowed, divorced, or separated showed a marginally stronger inverse association (OR = 0.97, 95% CI: 0.95–1.00, *P* = 0.035). Similarly, BMI did not exhibit significant interaction with VD levels (P for interaction = 0.870). However, the association appeared strongest in women with BMI ≥ 30 kg/m^2^ (OR = 0.98, 95% CI: 0.97–1.00, *P* = 0.033), suggesting a potentially greater sensitivity among individuals with obesity. No significant interactions were found for smoking status (*P* = 0.760), drinking status (*P* = 0.917), or hypertension (*P* = 0.726). Across these subgroups, the direction of association remained consistent, though effect sizes were modest and generally did not reach statistical significance. The results are shown in Figure 3.

Sensitivity analyses were conducted to assess the robustness of the findings. First, multivariable logistic regression analyses were repeated after excluding participants with major metabolic comorbidities, including hypertension and diabetes. Second, serum vitamin D was alternatively modeled using quartiles to evaluate whether the observed associations were sensitive to different exposure definitions. The overall direction and magnitude of the associations remained materially unchanged across all sensitivity analyses. The results are shown in Table 3.

**TABLE 3:** Sensitivity analyses.

	Model 1		Model 2		Model 3	
	OR (95% CI)	*P*-value	OR (95% CI)	*P*-value	OR (95% CI)	*P*-value
**PCOS**						
Serum vitamin D (continuous)	0.86 (0.81, 0.92)	< 0.001	0.87 (0.82, 0.93)	< 0.001	0.85 (0.79, 0.91)	< 0.001
<50 nmol/L	[Reference]		[Reference]		[Reference]	
≥50 nmol/L	0.45 (0.36, 0.56)	<0.001	0.44 (0.35, 0.55)	<0.001	0.42 (0.33, 0.54)	<0.001
P for trend	<0.001		<0.001		<0.001	
**Sensitivity analysis†**						
Excluding hypertension and diabetes	0.46 (0.36, 0.59)	< 0.001	0.45 (0.34, 0.58)	<0.001	0.43 (0.33, 0.57)	<0.001

Model 1: No covariates adjusted. Model 2: Adjusted for demographic and lifestyle factors, including age, educational level, marital status, smoking status, and alcohol consumption. Model 3: Further adjusted for reproductive history (pregnancy status), physiological indicators (body mass index, hypertension, and diabetes), and biochemical markers (C-reactive protein, total testosterone, and glutathione). †Sensitivity analysis was conducted by excluding participants with major metabolic comorbidities (hypertension and diabetes). CI, Confidence Interval; OR, Odds Ratio; VD, Vitamin D; PCOS, Polycystic Ovary Syndrome; BMI, Body Mass Index; CRP, C-reactive Protein; GSH, Glutathione.

## 4 Discussion

This study systematically evaluated the association between serum VD levels and the risk of PCOS in a large clinical population. We found that higher serum VD concentrations were independently associated with a lower risk of PCOS, and this association remained robust after accounting for demographic, lifestyle, and metabolic covariates. Notably, the protective effect of VD was particularly pronounced in older, obese, widowed/divorced/separated, and parous women, suggesting that specific endocrine–metabolic contexts may modify the biological response to VD.

Mechanistically, VD may exert a protective role in PCOS through several interrelated pathways. Binding of VD to VDR receptors on pancreatic β-cells enhances insulin secretion and peripheral glucose uptake, improving insulin sensitivity and reducing compensatory hyperinsulinemia—a central driver of ovarian androgen excess in PCOS (13). VD also exhibits anti-inflammatory and immunomodulatory effects by inhibiting NF-κB signaling and suppressing pro-inflammatory cytokine release, thus reducing the chronic low-grade inflammation characteristic of PCOS (14). In addition, VD influences reproductive endocrine regulation, modulating hypothalamic–pituitary–ovarian (HPO) axis function, lowering serum androgen levels, promoting follicular maturation, and restoring ovulatory cyclicity (15). These mechanisms collectively provide a biological basis for the observed reduction in PCOS risk across increasing VD levels.

Subgroup analyses revealed notable heterogeneity in the association between serum VD levels and PCOS risk. The protective effect of VD was strongest among obese women with BMI ≥ 30, older women aged ≥ 40 years, widowed/divorced/separated individuals, and women with previous pregnancy, with each 1 nmol/L increase in VD associated with a 2–3% reduction in PCOS risk. In contrast, no significant associations were observed among younger, normal-weight, never-married, or nulliparous women. The amplified protective effect observed in obese women is biologically plausible. Obesity alters VD distribution, storage, and metabolism (7). Excess adipose tissue promotes sequestration of fat-soluble VD, lowering its circulating bioavailability (16, 17). Moreover, obese individuals commonly exhibit chronic low-grade inflammation, insulin resistance, and increased secretion of pro-inflammatory adipokines, which synergistically exacerbate metabolic and endocrine disturbances in PCOS (18). Under these conditions, VD may exert stronger protective effects by improving insulin sensitivity, inhibiting inflammatory signaling pathways, and modulating hypothalamic–pituitary–ovarian (HPO) axis activity (19). Similarly, older and parous women may benefit more from adequate VD due to cumulative endocrine–metabolic fluctuations, altered sex hormone dynamics, and increased vulnerability to inflammation-related HPO axis disruption. These subgroup-specific findings are consistent with growing evidence that VD supplementation improves ovulatory frequency, reduces serum testosterone, and enhances fertility among women with PCOS, especially those who are obese or parous (13, 20, 21). Experimental studies further show that VD can downregulate ovarian CYP17A1 and other steroidogenic enzymes, correct insulin resistance–mediated hyperandrogenism, and alleviate inflammation-induced impairments in reproductive signaling, thereby improving reproductive outcomes. Overall, the subgroup results highlight that the physiological impact of VD on PCOS may vary according to adiposity, age, reproductive history, and metabolic–inflammatory burden. These findings underscore the importance of considering individual metabolic profiles when evaluating VD status and its potential role in PCOS prevention and management.

Restricted cubic spline analysis revealed a linear, dose–response relationship between serum VD levels and PCOS risk. No threshold or plateau effect was observed, suggesting that incremental increases in VD may continuously improve PCOS-related endocrine and metabolic dysregulation. This highlights the potential value of maintaining adequate VD status across the female reproductive lifespan. Our findings are generally consistent with previous studies reporting lower 25-hydroxyvitamin D levels among women with PCOS and strong associations with hyperandrogenism, dysovulation, and metabolic dysfunction (12, 22–24). Variability in the literature may be attributed to differences in sample size, diagnostic criteria, seasonal timing of VD measurement, or adjustment for confounders. The present study strengthens existing evidence by using standardized Rotterdam criteria, comprehensive covariate adjustment, and stratified subgroup analyses, thereby improving reliability and interpretability. Clinically, these results underscore the importance of monitoring and correcting VD deficiency as a cost-effective preventive or adjunctive strategy for PCOS. Women who are older, obese, or parous may represent high-yield targets for individualized supplementation, given their stronger biological response to higher VD levels. The observed dose–response pattern supports tailored approaches to maintaining sufficient VD concentrations to optimize reproductive and metabolic outcomes.

Nevertheless, this study has limitations. The retrospective design restricts causal inference. Serum VD was measured once, which may not fully capture long-term levels or seasonal fluctuations. Potential residual confounding from dietary habits, sunlight exposure, or genetic polymorphisms affecting VD metabolism may persist. In addition, participants were recruited from a single center, potentially limiting generalizability. Future research should incorporate multicenter prospective cohorts, repeated biomarker assessments, and mechanistic assays—including inflammatory, metabolic, and androgenic pathways—to refine causal understanding and inform targeted supplementation strategies.

In summary, higher serum VD levels are independently associated with a lower risk of PCOS, with protective effects particularly marked in older, obese, widowed/divorced/separated, and parous women. These findings highlight VD as a modifiable factor that may influence PCOS pathogenesis through metabolic, inflammatory, and endocrine mechanisms. Further prospective and interventional studies are warranted to determine optimal supplementation strategies and to clarify whether individualized VD optimization can improve reproductive and metabolic outcomes in high-risk subgroups.

## Data Availability

The raw data supporting the conclusions of this article will be made available by the authors, without undue reservation.

## References

[1] Joham AE Norman RJ Stener-Victorin E Legro RS Franks S Moran LJ Polycystic ovary syndrome. *Lancet Diabet Endocrinol.* (2022) 10:668–80. 10.1016/S2213-8587(22)00163-2 35934017

[2] Stener-Victorin E Teede H Norman RJ Legro R Goodarzi MO Dokras A Polycystic ovary syndrome. *Nat Rev.* (2024) 10:27. 10.1038/s41572-024-00511-3 38637590

[3] Teede HJ Tay CT Laven JJE Dokras A Moran LJ Piltonen TT Recommendations From the 2023 International evidence-based guideline for the assessment and management of polycystic ovary syndrome. *J Clin Endocrinol Metab.* (2023) 108:2447–69. 10.1210/clinem/dgad463 37580314 PMC10505534

[4] Sánchez-Garrido MA Serrano-López V Ruiz-Pino F Vázquez MJ Rodríguez-Martín A Torres E Superior metabolic improvement of polycystic ovary syndrome traits after GLP1-based multi-agonist therapy. *Nat Commun.* (2024) 15:8498. 10.1038/s41467-024-52898-y 39353946 PMC11445520

[5] Bouillon R Manousaki D Rosen C Trajanoska K Rivadeneira F Richards JB. The health effects of vitamin D supplementation: evidence from human studies. *Nat Rev Endocrinol.* (2022) 18:96–110. 10.1038/s41574-021-00593-z 34815552 PMC8609267

[6] Mohan A Haider R Fakhor H Hina F Kumar V Jawed A Vitamin D and polycystic ovary syndrome (PCOS): a review. *Ann Med Surg.* (2023) 85:3506–11. 10.1097/MS9.0000000000000879 37427232 PMC10328709

[7] Wen X Wang L Li F Yu X. Effects of vitamin D supplementation on metabolic parameters in women with polycystic ovary syndrome: a randomized controlled trial. *J Ovarian Res.* (2024) 17:147. 10.1186/s13048-024-01473-6 39014475 PMC11251207

[8] Cochrane KM Bone JN Williams BA Karakochuk CD. Optimizing vitamin D status in polycystic ovary syndrome: a systematic review and dose-response meta-analysis. *Nutr Rev.* (2024) 82:1176–86. 10.1093/nutrit/nuad117 37769789

[9] Rotterdam Eshre/Asrm-Sponsored Pcos consensus workshop group. Revised 2003 consensus on diagnostic criteria and long-term health risks related to polycystic ovary syndrome (PCOS). *Hum Reprod.* (2004) 19:41–7. 10.1093/humrep/deh098 14688154

[10] Ma J Li K. Negative association between serum vitamin D levels and depression in a young adult US population: a cross-sectional study of NHANES 2007-2018. *Nutrients.* (2023) 15:2947. 10.3390/nu15132947 37447273 PMC10346331

[11] Discacciati A Palazzolo MG Park JG Melloni GEM Murphy SA Bellavia A. Estimating and presenting non-linear associations with restricted cubic splines. *Int J Epidemiol.* (2025) 54:dyaf088. 10.1093/ije/dyaf088 40527479

[12] Shan C Zhu Y-C Yu J Zhang Y Wang Y-Y Lu N Low serum 25-hydroxyvitamin D levels are associated with hyperandrogenemia in polycystic ovary syndrome: a cross-sectional study. *Front. Endocrinol.* (2022) 13:894935. 10.3389/fendo.2022.894935 35586624 PMC9108253

[13] Gao B Zhang C Wang D Li B Shan Z Teng W Causal association between low vitamin D and polycystic ovary syndrome: a bidirectional mendelian randomization study. *J Ovarian Res.* (2024) 17:95. 10.1186/s13048-024-01420-5 38715063 PMC11077756

[14] Yang M Shen X Lu D Peng J Zhou S Xu L Effects of vitamin D supplementation on ovulation and pregnancy in women with polycystic ovary syndrome: a systematic review and meta-analysis. *Front Endocrinol.* (2023) 14:1148556. 10.3389/fendo.2023.1148556 37593349 PMC10430882

[15] Zhang B Yao X Zhong X Hu Y Xu J. Vitamin D supplementation in the treatment of polycystic ovary syndrome: a meta-analysis of randomized controlled trials. *Heliyon.* (2023) 9:e14291. 10.1016/j.heliyon.2023.e14291 36942243 PMC10023924

[16] Park CY Han SN. Vitamin D and obesity. *Adv Food Nutr Res.* (2024) 109:221–47. 10.1016/bs.afnr.2023.12.006 38777414

[17] Li R Li Z Huang Y Hu K Ma B Yang Y. The effect of magnesium alone or its combination with other supplements on the markers of inflammation, OS and metabolism in women with polycystic ovarian syndrome (PCOS): a systematic review. *Front Endocrinol.* (2022) 13:974042. 10.3389/fendo.2022.974042 35992132 PMC9389579

[18] Kohlhoff G Kirwan R Mushtaq S. The effect of vitamin D supplementation on markers of insulin resistance in women with polycystic ovarian syndrome: a systematic review. *Eur J Nutr.* (2024) 63:2859–69. 10.1007/s00394-024-03489-6 39276209 PMC11519308

[19] Lee MJ. Vitamin D enhancement of adipose biology: implications on obesity-associated cardiometabolic diseases. *Nutrients.* (2025) 17:586. 10.3390/nu17030586 39940444 PMC11820181

[20] Piao C Li J Liang C Zhang J Li X Zhao Z Effect of vitamin D on pregnancy in women with polycystic ovary syndrome: retrospective and prospective studies. *Reprod Biomed Online.* (2024) 49:103909. 10.1016/j.rbmo.2024.103909 38776748

[21] Asemi Z. Vitamin D and probiotic co-supplementation affects mental health, hormonal, inflammatory and oxidative stress parameters in women with polycystic ovary syndrome. *J Ovarian Res.* (2019) 12:5. 10.1186/s13048-019-0480-x1186/s13048-024-01420-5 30665436 PMC6340184

[22] Zerrouki D Rami I Assarrar I Bouichrat N Rouf S Latrech H. Is there any association between vitamin D status and PCOS disease? *Gynecol Endocrinol.* (2024) 40:2381501. 10.1080/09513590.2024.2381501 39481002

[23] Mesinovic J Teede HJ Shorakae S Lambert GW Lambert EA Naderpoor N The relationship between vitamin D metabolites and androgens in women with polycystic ovary syndrome. *Nutrients.* (2020) 12:1219. 10.3390/nu12051219 32357490 PMC7282251

[24] Kazeminia M Rajati F Rasulehvandi R Rajati M. The effect of vitamin D on the hormonal profile of women with polycystic ovarian syndrome: a systematic review and meta-analysis. *Middle East Fertility Soc J.* (2024) 29:45. 10.1186/s43043-024-00201-w

